# Spin in Published Reports of Tinnitus Randomized Controlled Trials: Evidence of Overinterpretation of Results

**DOI:** 10.3389/fneur.2021.693937

**Published:** 2021-07-16

**Authors:** Hedwig M. Velde, Jan A. A. van Heteren, Adriana L. Smit, Inge Stegeman

**Affiliations:** ^1^Department of Otorhinolaryngology, Head and Neck Surgery, University Medical Center Utrecht, Utrecht, Netherlands; ^2^University Medical Center Utrecht Brain Center, University Medical Center Utrecht, Utrecht, Netherlands; ^3^Department of Ophthalmology, University Medical Center Utrecht, Utrecht, Netherlands; ^4^Epidemiology and Data Science, Amsterdam University Medical Center, University of Amsterdam, Amsterdam, Netherlands

**Keywords:** tinnitus, methods, quality, SPIN, randomized controlled trial

## Abstract

**Background:** Spin refers to reporting practices that could distort the interpretation and mislead readers by being more optimistic than the results justify, thereby possibly changing the perception of clinicians and influence their decisions. Because of the clinical importance of accurate interpretation of results and the evidence of spin in other research fields, we aim to identify the nature and frequency of spin in published reports of tinnitus randomized controlled trials (RCTs) and to assess possible determinants and effects of spin.

**Methods:** We searched PubMed systematically for RCTs with tinnitus-related outcomes published from 2015 to 2019. All eligible articles were assessed on actual and potential spin using prespecified criteria.

**Results:** Our search identified 628 studies, of which 87 were eligible for evaluation. A total of 95% of the studies contained actual or potential spin. Actual spin was found mostly in the conclusion of articles, which reflected something else than the reported point estimate (or CI) of the outcome (*n* = 34, 39%) or which was selectively focused (*n* = 49, 56%). Linguistic spin (“trend,” “marginally significant,” or “tendency toward an effect”) was found in 17% of the studies. We were not able to assess the association between study characteristics and the occurrence of spin due to the low number of trials for some categories of the study characteristics. We found no effect of spin on type of journal [odds ratio (OR) −0.13, 95% CI −0.56–0.31], journal impact factor (OR 0.17, 95% CI −0.18–0.51), or number of citations (OR 1.95, CI −2.74–6.65).

**Conclusion:** There is a large amount of spin in tinnitus RCTs. Our findings show that there is room for improvement in reporting and interpretation of results. Awareness of different forms of spin must be raised to improve research quality and reduce research waste.

## Introduction

Randomized controlled trials (RCTs) are considered to have the highest level of evidence for assessing the effects of clinical interventions (1) and therefore are the gold standard to study the safety and efficacy of new treatments (2). The accurate presentation of the results of RCTs is the cornerstone of the dissemination of the results and their implementation in clinical practice (3). Therefore, the expanding number of reports about research waste due to the incorrect use of research methods and biased reporting of results is worrisome (4).

“Spin” is a phenomenon that refers to reporting practices that could distort the interpretation of study results and mislead readers by a more optimistic presentation than justified (3, 5). It was first described in 2007 by Fletcher and Black (6) who stated that scientific results published in medical journals are not simply the recitation of facts, following an original protocol and objective data, but rather the reflection of a complex set of social forces that might distort the message. In the literature, it is also described as overinterpretation, misrepresentation, or misreporting of results (7, 8). Spin may change the perception of clinicians and influence their decisions, especially when results of RCTs become integrated in treatment recommendations and guidelines. If the reporting of research outcomes is “spinned,” readers are more likely to rate a treatment as beneficial despite, for example, a statistically non-significant outcome (9).

Since the first systematic assessment by Boutron et al. (3) in 2010, spin has been studied in diagnostic accuracy studies (7), RCTs (10, 11), and prognostic factor studies (12), with various clinical specialties such as psychiatry (13) or acupuncture (14). Their conclusions match: spin frequently occurs in different areas of medical research.

Tinnitus is a common condition for which, to date, an effective personalized treatment remains to be found. Several methodological and reporting issues have been identified that hinder the findings of a curative treatment. To improve the quality of tinnitus-research, we aim to identify the nature and frequency of spin in published reports of tinnitus RCTs. The secondary aim is to assess possible determinants and effects of spin. Outcomes will contribute to an improvement of research quality and reduce research waste by creating awareness of different forms of bias and spin in the reporting of tinnitus results.

## Methods

### Literature Search

We searched the literature in PubMed on October 18, 2019, for all RCTs with tinnitus-related outcomes published from 2015 up to October 18, 2019 (see Appendix 1 for the search strategy). Two authors (HV, IS) independently reviewed the titles and abstracts of the identified articles. We included articles that were RCTs reporting on efficacy and/or safety of interventions for tinnitus, with tinnitus severity (burden/impact/distress) as primary outcome measure (e.g., Tinnitus Handicap Inventory, Tinnitus Severity Questionnaire, Visual Analog Scale). Subsequently, the remaining articles were independently assessed on eligibility in full text. The search was complemented by checking reference lists. All disagreements were resolved by discussion.

### Data Extraction and Assessment of Variables

One author (HV) extracted the following data from the included RCTs: first author, date of publication and date of online access, continent where the study was conducted, journal of publication, journal impact factor in the year of publication (15), journal type (“specialty ENT,” “general medical,” or “other”), and number of citations (16).

The time since publication was calculated as the time in months from date of online access until December 19, 2020. If the date of online access was unknown, we used the 15th of the month of the printed publication.

The following data were extracted and assessed by two authors independently (HV, IS): type of experimental and comparative intervention, whether the study focused on safety or efficiency of an intervention, whether the primary outcome was statistically significant or not, the degree of positivity of the conclusion in the abstract and in the full text, source of funding, disclosure of conflict of interest, use of a reporting guideline, whether or not the trial was registered in a trial database, and whether or not a power analysis was conducted.

For the type of experimental and comparative intervention, the following categories were used: “neuromodulation,” “drug,” “psychoeducational intervention,” “device,” “other,” “>1 intervention category.” Additionally, the type of comparative intervention included the categories “placebo/sham,” “usual care,” and “no care.” For the type of primary outcome (i.e., efficacy and/or safety), we assessed what was described in the abstract or introduction as the aim of the study.

The results of the primary outcomes were considered statistically significant if they were reported as such by the authors [i.e., “statistically significant,” a *p*-value <0.05, or if the 95% confidence interval (95% CI) around the observed effect size excluded the no-effect value]. Consequently, we scored the primary outcome as “significant,” “non-significant,” “both” (in case of multiple primary outcomes), or “not applicable” (in case of no testing of between-group differences).

To determine the positivity of the conclusion in the abstract and in the main text, we applied an adapted form of the assessment as reported by McGrath et al. (5). For all included studies, we determined whether the conclusion was “positive,” “neutral,” or “negative” or noted “no conclusion” if no conclusion was reported.

We categorized funding as “not for profit,” “for profit,” “mixed,” “no funding,” or “not reported.” Whether or not a reporting guideline was used, the trial was registered in a clinical trial database, or a power analysis was performed was scored as “yes” or “not reported” based on what was reported by the authors in the article. Note that we did not check on the accuracy of following a reporting guideline.

### Assessment of Spin

Spin criteria were composed by three authors (HV, IS, DS) based on two studies (3, 5). McGrath et al. (5) published a list of actual and potential overinterpretation criteria in systematic reviews of diagnostic accuracy studies. We discarded the criteria aimed at systematic reviews and/or diagnostic accuracy studies and adjusted these criteria for the use in therapeutic studies by supplementing criteria as reported by Boutron et al. (3) who focused on RCTs. As Boutron et al. (3) only investigated studies with statistically non-significant outcomes, these criteria were adjusted to be used also in studies with statistically significant outcomes. In the initial modification process, eight of the original criteria were discarded because they were not subject to the scope of our study and one criterion (unclear conflict of interest) was discarded but included in the study characteristics.

The provisional modified list of criteria for spin was independently tested by two authors (HV, IS) on five randomly selected studies from our study sample to determine to what extent the assessment of the researchers matched and to further evaluate the criteria. In general, there was consensus. However, two additional criteria have been discarded in that process. For one criterion (conclusion not taking high risk of bias and/or applicability concerns into account), this was due to a lack of objectivity in the assessment of the included studies. For the other criterion (no or inadequate assessment of risk of bias and applicability concerns), it was due to the lack of discriminatory value, as discussing the risk of bias concerns or applicability concerns does not have to say anything about the quality of that assessment. Therefore, in our opinion, an unjustified better score was achieved by, for example, studies that addressed their limitations inadequately compared to studies that did not address their limitations. The final list of actual and potential spin criteria with, where relevant, examples of how these criteria were scored is provided in Appendix 2. The modification process of the criteria is shown in Appendix 3.

Actual spin was defined as a conclusion not reporting the point estimate(s) of outcome(s), a selectively focused or extrapolated conclusion, a stronger conclusion in abstract than in full text, and/or linguistic spin (i.e., “trend,” “tendency toward,” or “marginally significant/approaching significance”). Potential spin refers to practices that facilitate overinterpretation but make a formal assessment impossible (5). The presence of potential spin was defined as no reporting of the point estimate or the CI or the *p*-value or the standard deviation (SD) in the abstract and the full text and/or not discussing study limitations.

Two authors (HV, IS) independently assessed and scored actual and potential spin in the included articles. Disagreements were resolved by discussion.

### Data Analysis

The primary outcome was the nature and frequency of actual and potential spin. Descriptive statistics were used to report on the primary outcome. Secondary outcomes were (1) the association between study characteristics and the occurrence of spin and (2) the effects of spin on type of journal, journal impact factor, and number of citations. Linear regression analyses were performed for the secondary outcomes. We considered a 95% CI not containing the value 0.00 and a *p*-value <0.05 as statistically significant. Statistical analyses were completed using SPSS software (version 25, IBM Corp., Armonk, NY, USA).

## Results

### Search Results

**Figure 1:**
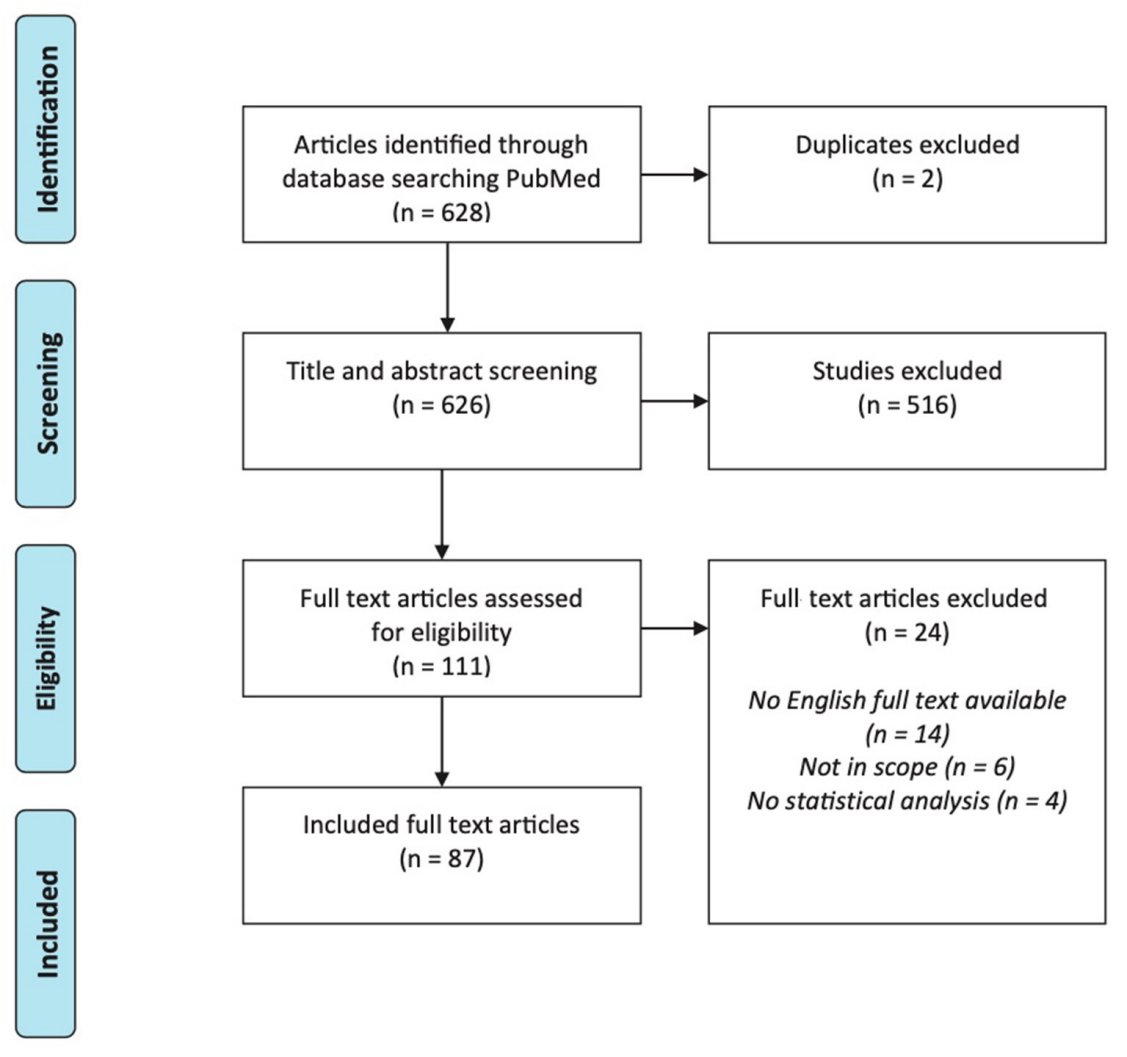
PRISMA flow diagram representing the search and screening process.

Our search identified 628 articles in PubMed (Figure 1). After title and abstract screening, 111 articles were selected for full-text screening. Of these, 87 interventional tinnitus studies met all eligibility criteria.

### Characteristics of Included Studies

Most included RCTs were conducted in Europe (*n* = 28, 32%), North America (*n* = 22, 25%), or Asia (*n* = 19, 22%) (Table 1). Neuromodulation was the most frequently studied experimental intervention (*n* = 26, 30%) for tinnitus. Experimental interventions were mostly compared to placebo or sham (*n* = 38, 44%). Most studies investigated the efficacy of the intervention (*n* = 84, 97%). One study (1%) focused on safety, and two (2%) studies investigated both efficacy and safety. In 28 studies (32%) the difference in primary outcome between the intervention and the control group was statistically significant, and in half of the studies, the difference in outcome was not statistically significant (*n* = 43, 49%). In about two-thirds of the studies, the conclusion in the abstract and full text was positive (*n* = 60, 69% and *n* = 59, 68%, respectively, in the abstract and full text). The most common funding source was non-profit organizations (*n* = 52, 60%), and in 55 articles (63%), there were no conflicts of interest reported. Most studies (*n* = 67, 77%) did not report on following a report guideline. Trial registration and power analysis were reported in almost half of the included trials (*n* = 43, 49%, and *n* = 37, 43%, respectively). All study characteristics are summarized in Table 1.

**Table 1:** Study characteristics.

**Variables**	**Value**	**Number of studies (%)**
Year of publication	2015	14 (16.1)
	2016	25 (28.7)
	2017	21 (24.1)
	2018	16 (18.4)
	2019	11 (12.6)
Geographic distribution	Africa	3 (3.4)
	Asia	19 (21.8)
	Australia	5 (5.7)
	Europe	28 (32.2)
	North America	22 (25.3)
	South America	7 (8.0)
Experimental intervention	Neuromodulation	26 (29.9)
	Drug	19 (21.8)
	Psychoeducational intervention	13 (14.9)
	Device	14 (16.1)
	Other	11 (12.6)
	>1 experimental intervention category	4 (4.6)
Comparative intervention	Placebo/sham	38 (43.7)
	Usual care	5 (5.7)
	No care	6 (6.9)
	Drug	5 (5.7)
	Psychoeducational intervention	6 (6.9)
	Neuromodulation	7 (8.0)
	Device	12 (13.8)
	Other	4 (4.6)
	>1 comparative intervention category	4 (4.6)
Type of primary outcome(s)	Efficacy	84 (96.6)
	Safety	1 (1.1)
	Both	2 (2.3)
Statistical significance of primary outcome(s)	Non-significant	43 (49.4)
	Significant	28 (32.2)
	Both (in case of multiple primary outcomes)	7 (8.0)
	N/A (no testing of between group differences)	9 (10.3)
Positivity of conclusion in abstract^$^	Positive	60 (69.0)
	Neutral	7 (8.0)
	Negative	18 (20.7)
	No abstract or no conclusion in abstract	2 (2.3)
Positivity of conclusion in full text^$^	Positive	59 (67.8)
	Neutral	8 (9.2)
	Negative	19 (21.8)
	No conclusion in full text	1 (1.1)
Funding	Not for profit	52 (59.8)
	For profit	6 (6.9)
	Mixed	4 (4.6)
	No funding	5 (5.7)
	Not reported	20 (23.0)
Conflict of interest	Yes	13 (14.9)
	No	55 (63.2)
	Not reported	19 (21.8)
Use of reporting guideline	Yes, CONSORT checklist	8 (9.2)
	Yes, CONSORT flow diagram	12 (13.8)
	Not reported	67 (77.0)
Trial registration	Yes	43 (49.4)
	Not reported	44 (50.6)
Power analysis	Yes	37 (42.5)
	Not reported	50 (57.5)

$ *Examples of how positivity of conclusions were scored are published in Appendix 3*.

*N/A, not applicable*.

### Assessment of Spin

In four (5%) of the included RCTs, no actual or potential spin was reported. Sixty-five (75%) studies contained one or more forms of actual spin, and 74 (85%) studies contained one or more forms of potential spin.

### Actual Spin

In 34 (39%) of all included studies, the conclusion did not reflect the reported point estimate of the outcome (Table 2). In 49 (56%) articles, authors had a selective focus on the results other than the comparison between the arms in an RCT. In most cases, the focus was on within-group differences instead of between-group differences (*n* = 40, 46%). The conclusion was inappropriately extrapolated to a wider population or setting and/or extrapolated as surrogates for improvement in patient important outcomes in 18 (21%) articles. In 11 (13%) articles, the conclusion in the abstract was stronger than the conclusion in the full text. Linguistic spin was present in 15 (17%) articles by using the words or phrases “trend” (*n* = 10, 67% of total linguistic spin), “marginally significant/approaching significance” (*n* = 3, 20%), and “tendency toward a decrease/an effect” (*n* = 2, 13%).

**Table 2:** Nature and frequency of actual and potential spin.

**Actual spin**	***N* (%)**
**Conclusion reflecting anything other than the reported point estimate (and CI) of outcome**	
No	53 (60.9)
Yes	34 (39.1)
**Selectively focused conclusion**	
No	38 (43.7)
Within-group comparison	40 (46.0)
Secondary outcome	2 (2.3)
Subgroup analysis	1 (1.1)
Modified population of analyses	0 (0.0)
Focused on one arm	5 (5.7)
Other^*^	1 (1.1)
**Conclusion inappropriately extrapolated to a wider population or setting**	
No	73 (83.9)
Yes	14 (16.1)
**Conclusion inappropriately extrapolated as surrogates for improvement in patient important outcomes**	
No	83 (95.4)
Yes	4 (4.6)
**Stronger conclusion in abstract than in full text**	
No	75 (86.2)
Yes	11 (12.6)
No abstract	1 (1.1)
**Linguistic spin**	
No	72 (82.8)
Trend	10 (11.5)
Marginally significant/approaching significance	3 (3.4)
Tendency toward a decrease/an effect	2 (2.3)
**Potential spin**	
**Reporting of point estimate(s) of the outcome(s) in the abstract**	
Yes	26 (29.9)
No	60 (69.0)
No abstract	1 (1.1)
**CIs around point estimates in the abstract**	
Yes	10 (11.5)
No	16 (18.4)
No point estimate in abstract	60 (69.0)
No abstract	1 (1.1)
***P*****-value in abstract, in case of absence of CI in abstract**	
Yes	27 (31.0)
No	8 (9.2)
No point estimate in abstract	44 (50.6)
CI of point estimate in abstract	7 (8.0)
No abstract	1 (1.1)
**SD in abstract, in case of absence of CI in abstract**	
Yes	3 (3.4)
No	13 (14.9)
No point estimate in abstract	60 (60.9)
CI of point estimate in abstract	10 (11.5)
No abstract	1 (1.1)
**Reporting of point estimate of the outcome in the full text**	
Yes	77 (88.5)
No	10 (11.5)
**CIs around point estimates in the full text**	
Yes	17 (19.5)
No	60 (69.0)
No point estimate in full text	10 (11.5)
***P*****-value in full text, in case of absence of CI in full text**	
Yes	70 (80.5)
No	4 (4.6)
No point estimate in full text	5 (5.7)
CI of point estimate in full text	8 (9.2)
**SD in full text, in case of absence of CI in full text**	
Yes	48 (55.2)
No	21 (24.1)
No point estimate in full text	9 (10.3)
CI of point estimate in full text	9 (10.3)
**Discussion of study limitations**	
Yes	53 (60.9)
No	34 (39.1)

* *Focus on other question than research question*.

### Potential Spin

In 60 (69%) articles, there was no reporting of the point estimate in the abstract (Table 2). If reporting a point estimate, in 16 (18% of total) articles, there was no CI reported. In the absence of a CI, eight (9%) articles also reported no *p*-value and 13 (15%) articles no SD. The point estimate was not reported in the full text of 10 (12%) articles. In 60 (69% of total) of the articles with a reported point estimate, no CI was reported. In that case, four (5%) articles also reported no *p*-value and 21 (24%) articles also no SD. In 34 (39%) articles, there was no discussion of study limitations.

### Secondary Outcomes

We were not able to analyze the association between study characteristics and the occurrence of spin because of the low number of papers without spin (Table 1).

Most studies were published in ENT/otorhinolaryngology journals (*n* = 52, 60%), 11 studies (13%) were published in general medical journals, and 24 studies (28%) in other journals, mainly in the field of neurology and radiology (Table 3). The mean journal impact factor in the year of publication was 1.16 (SD = 0.70), and the mean number of citations corrected for time since publication was 10.43 (SD = 9.62). The presence of actual spin was not found to be a statistically significant determinant of the type of the journal that published the article [odds ratio (OR) −0.13, 95% CI −0.56–0.31], the mean journal impact factor (OR 0.17, 95% CI 0.18–0.51), or the number of citations of the article after publication (OR 1.95, CI −2.74–6.65).

**Table 3:** Effects of actual spin.

**Variables**	***N* (%)/mean (SD)**	**B (95%CI)**	***P*-value**
Type of journal		−0.13 (−0.56–0.31)	0.56
Specialty ENT	52 (59.8)		
General medical	11 (12.6)		
Other	24 (27.6)		
Journal impact factor		0.17 (−0.18–0.51)	0.34
Spin	1.21 (±0.72)		
No-spin	1.04 (±0.61)		
Number of citations^*^		1.95 (−2.74–6.65)	0.41
Spin	10.91 (±9.88)		
No spin	9.14 (±8.96)		

* *Adjusted for time since publication in months*.

*CI, confidence interval; ENT, ear-nose-throat; SD, standard deviation; OR, odds ratio*.

## Discussion

The main goal of this study was to identify the nature and frequency of actual and potential spin in published reports of tinnitus RCTs. Our results show that 95% of all included studies contained actual or potential spin. We were not able to assess the association between study characteristics and the occurrence of spin due to the low number of trials without spin. We found no relation between spin and type of journal, journal impact factor, and number of citations.

The high amount of spin we found in our study may have implications for clinical practice. A recent review on biomarker studies showed that highly cited studies often overestimated the findings of meta-analyses (17). Overinterpretation, as represented by spin, is responsible for potentially harmful clinical decisions (9). Also, in an era of evidence-based medicine, where guidelines are crucial for clinical practice, spin in primary studies might be the start of a cascade of suboptimal decisions, where first the outcomes of the spin-containing study is wrongfully used in guidelines and thereby used by clinicians.

Our sample size (*n* = 87) was comparable to other spin studies with sample sizes of 100 to 150 articles (5). However, the amount of spin we found is higher than described in previous studies (3, 5, 7, 8, 10–12, 18–22).

Spin has its implications for research and therefore can hinder us in finding a treatment for tinnitus. Optimal research methods, design, analysis, and reporting are the fundamentals of biomedical research. Tinnitus research is a relatively young field; to date, 11,000 studies on tinnitus have been published on PubMed. A wide variety of medical and paramedical specialties are involved in the clinical care for tinnitus patients as well as in research. This combination of clinical and methodological knowledge has enormous advantages for both fields. Over the past decade, tinnitus researchers worldwide have made important steps forward, starting with global collaborations where quality of research plays an important role (23, 24). Such initiatives have the potential to lead to quality improvements, resulting in more effective research and a better path to finding an effective treatment.

Reducing spin and its effects is the responsibility of everyone involved in biomedical research, including authors, peer reviewers, and journal editors. Ignorance of scientific standards, young researchers imitating previous practice, unconscious prejudice, or willful intent to influence readers may be responsible for the presence of spin (25). Several interventions for improving the quality of biomedical research have been implemented over the years. Writing guidelines can improve the completeness of reporting results (25). One might argue that such guidelines can also provide information about writing research results in an objective manner.

Several methodological considerations need to be considered when drawing conclusions about the results of our study. For the assessment of spin, we were limited to what was reported by the authors of the included studies. In some cases, the description of the study, its results, or its methods were suboptimal. We then made assumptions about, for example, the primary outcome of the study. Also, despite the blind assessment of two authors, assessing spin has its subjective components. Although our list of spin criteria was based on previous studies, it was customized, which could have led to cognitive bias. This is the case for all self-developedcriteria lists in research investigating spin. The results of different spin studies would be better comparable if a uniform list of criteria was used. Lastly, to optimize the objectivity of the data we extracted, we chose to only focus on whether something was reported and not on the substantive accuracy. For example, we chose to only score whether study limitations were discussed and not if that discussion was sufficient. Also, we found studies in which the calculation of the outcome measures was unclear but wherein the point estimates and CIs were reported (26). For the purpose of this study, we chose to only score whether those values were reported and not if they were, to our opinion, correct. When we consider the strengths of our study, we think that (1) we performed a complete and comprehensive literature review and (2) we used explicit, predefined criteria for spin based on two previous studies (3, 5). With an overview of how the criteria were modified and scored (Appendices 2 and 3), we aimed to optimize reproducibility and transparency.

To conclude, the findings of this and previous studies show that there is a considerable amount of spin in RCTs. Spin may change clinicians' perception and influence their decisions, especially when RCT results are integrated in treatment recommendations and guidelines. Awareness of different forms of bias and spin in the reporting of results is therefore important for clinical practice and tinnitus research. Hopefully, this will contribute to the search for cure for tinnitus. The common advise to authors is to adhere to reporting guidelines in general (e.g., CONSORT guidelines for RCTs) and omit any form of spin in their articles (27). Readers should be critical when reading articles and recognize forms of spin, especially in the abstract and conclusion of publications (10, 19, 20).

## Data Availability Statement

The raw data supporting the conclusions of this article will be made available by the authors, without undue reservation.

## Author Contributions

HV, AS, JH, and IS contributed to conception and design of the study and wrote sections of the manuscript. HV organized the database and wrote the first draft of the manuscript. HV and IS performed the statistical analysis. All authors contributed to manuscript revision, read, and approved the submitted version.

## Conflict of Interest

The authors declare that the research was conducted in the absence of any commercial or financial relationships that could be construed as a potential conflict of interest.
