# Supplementary material for: Spin in Published Reports of Tinnitus Randomized Controlled Trials: Evidence of Overinterpretation of Results
Source: Front Neurol. 2021 Jul 16;12:693937. doi: 10.3389/fneur.2021.693937 (PMC8322656; doi:10.3389/fneur.2021.693937)
Supplement: Supplementary file 1 [file Table_1.docx]

**Appendix 1. Search strategy (date of search: October 18, 2019)**

| **Search** | **Syntax** | **Results (n)** |
| --- | --- | --- |
| #1 | *Search syntax for RCT’s on Pubmed* | 2,327,099 |
|  | (((Randomized Controlled Trial[ptyp])) OR ((Controlled Clinical Trial[ptyp])) OR ((Clinical Trial[ptyp])) OR ("Clinical Trials as Topic"[Mesh]) OR ("Clinical Trials, Phase III as Topic"[Mesh]) OR ("Clinical Trials, Phase IV as Topic"[Mesh]) OR ("Controlled Clinical Trials as Topic"[Mesh]) OR ("Clinical Trial"[Publication Type]) OR ("Controlled Clinical Trial"[Publication Type]) OR ("Clinical Trial, Phase III"[Publication Type]) OR ("Clinical Trial, Phase IV"[Publication Type]) OR ("Multicenter Study"[Publication Type]) OR ("Multicenter Studies as Topic"[Mesh]) OR ("Random Allocation"[Mesh]) OR ("Double-Blind Method"[Mesh]) OR ("Single-Blind Method"[Mesh]) OR ("Cross-Over Studies"[Mesh]) OR ("Placebos"[Mesh]) OR (controlled[tiab] AND (trial[tiab] OR trials[tiab] OR study[tiab] OR studies[tiab])) OR (blind[tiab] OR blinding[tiab] OR blinded[tiab] OR mask[tiab] OR masking[tiab] OR masked[tiab] OR placebo[tiab] OR placebos[tiab] OR rct[tiab] OR random[tiab] OR randomised[tiab] OR randomized[tiab] OR randomly[tiab] OR randomisation[tiab] OR randomization[tiab]) OR (factorial[tiab]) OR (divided[tiab] AND (group[tiab] OR groups[tiab])) OR (crossover[tiab]) OR ("cross over"[tiab]) OR (multicentre[tiab] OR multicentred[tiab] OR multicentric[tiab]) OR (versus[ti] OR vs[ti]) OR ("treatment arm"[tiab]) OR ("phase III"[tiab] OR "phase three"[tiab] OR "phase 3"[tiab]) OR ("latin square"[tiab]) NOT (("Animals"[Mesh] OR mouse[ti] OR mice[ti] OR pig[ti] OR pigs[ti] OR rat[ti] OR rats[ti] OR rabbit*[ti]) NOT (("Animals"[Mesh] OR mouse[ti] OR mice[ti] OR pig[ti] OR pigs[ti] OR rat[ti] OR rats[ti] OR rabbit*[ti] OR cadaver[ti] OR cadavers[ti]) AND ("Humans"[Mesh])))) |  |
| #2 | *Search syntax for Tinnitus specified on PubMed* | 12,625 |
|  | ("tinnitus"[tiab] OR "tinnitus"[MeSH Terms]) |  |
| #3 | *Search syntax for date restriction* | 5,624,667 |
|  | (“2015”[Date – Publication] : “2019”[Date – Publication]) |  |
| #4 | *Search syntax to exclude publication types* | 1,953,554 |
|  | (letter[pt] OR comment[pt] OR editorial[pt] OR news[pt]) |  |
| #5 | *Search strategy* | 628 |
|  | (#1 AND #2 AND #3) NOT #4 |  |
