# Supplementary material for: Spin in Published Reports of Tinnitus Randomized Controlled Trials: Evidence of Overinterpretation of Results
Source: Front Neurol. 2021 Jul 16;12:693937. doi: 10.3389/fneur.2021.693937 (PMC8322656; doi:10.3389/fneur.2021.693937)
Supplement: Supplementary file 2 [file Table_2.docx]

**Appendix 2. Scoring of actual and potential spin**

| Variable | Label | Values | Spin if condition is satisfied |
| --- | --- | --- | --- |
| 1 | Conclusion, not reflecting the reported point estimate (and CI) of outcome | No; yes | 1=yes |
|  | *Example: No significant difference was found between experimental and comparative group, but in conclusion it is stated that one group scored better than the other.* | | |
| 2 | Selectively focused conclusion | No; within group comparison; secondary outcome; subgroup analyses; modified population of analyses; focused on one arm while more arms were analyzed; other | 2≠no |
|  | *Examples:*  *Within group comparison: No significant difference was found between experimental and comparative group, in the conclusion it is only stated that the experimental group scored better before than after treatment.*  *Secondary outcome: No significant difference was found between experimental and comparative group on the primary outcome as published in the article, in the conclusion it is only stated that the experimental intervention scored significantly better on a secondary outcome.*  *Subgroup analyses: No significant difference was found between experimental and comparative group in the overall study population, in the conclusion it is only stated that a statistically significant difference was found in a subgroup.*  *Modified population of analyses: a different (not defined) subgroup of patients was analysed*  *Focused on one arm while more arms were analyzed: More than one experimental subgroup of a type of intervention was included, the conclusion only focused on the group that scored significantly better than the comparative group.* | | |
| 3 | Conclusion, inappropriately extrapolated to a wider population or setting | No; yes | 3=yes |
|  | *Example: The study population consisted of a specific group, but the conclusion is not limited to that group.* | | |
| 4 | Conclusion, inappropriately extrapolated as surrogates for improvement in patient important outcomes | No; yes | 4=yes |
|  | *Example: In the conclusion it is stated that an intervention can improve the quality of life, while the primary outcome was the score on a symptom-specific questionnaire.* | | |
| 5 | Stronger conclusion in abstract than full text | No; yes; N/A (no abstract) | 5=yes |
|  | *Example: In the abstract conclusion it is stated that the experimental group scored better than the comparative group and only in the full text conclusion it is mentioned that the difference was not statistically significant.* | | |
| 6 | Linguistic spin | No; yes | 6=yes |
|  | *Example: No significant difference was found between experimental and comparative group, in the conclusion it is stated that there is a trend towards statistical significance.* | | |
| Total scoring of actual spin criteria: Act_1=yes \| Act_2≠no \| Act_3=yes \| Act_4=yes \| Act_5=yes \| Act_6=yes | | | |

| Name | Label | Values | Spin if condition is satisfied |
| --- | --- | --- | --- |
| 1 | No reporting of point estimate in abstract | No; yes; N/A (no abstract) | 1=yes |
| 2 | No CIs around point estimates of outcome in abstract | No; yes; N/A (no point estimate in abstract); N/A (no abstract) | 2=yes **+**  3=yes **+**  4=yes |
| 3 | No P-value in abstract, in case of absence of CI in abstract | No; yes; N/A (no point estimate in abstract); N/A (CI of point estimate in abstract); N/A (no abstract) |  |
| 4 | No SD in abstract, in case of absence of CI in abstract | No; yes; N/A (no point estimate in abstract); N/A (CI of point estimate in abstract); N/A (no abstract) |  |
| 5 | No reporting of point estimate in full text | No; yes | 5=yes |
| 6 | No CIs around point estimates of outcome in full text | No; yes; N/A (no point estimate in FT) | 6=yes **+**  7=yes **+**  8=yes |
| 7 | No P-value in full text, in case of absence of CI in abstract | No; yes; N/ A (no point estimate in FT); N/A (CI of point estimate in FT) |  |
| 8 | No SD in full text, in case of absence of CI in abstract | No; yes; N/A (no point estimate in FT); N/A (CI of point estimate in FT) |  |
| 9 | No study limitations discussed  *Regardless of the quality of the assessment of limitations.* | No; yes | 9=yes |
| Total scoring of potential spin criteria: 1=yes \| (2=yes + 3=yes + 4=yes) \| 5=yes \| (6=yes + 7=yes + 8=yes) \| 9=yes | | | |
