# Supplementary material for: Spin in Published Reports of Tinnitus Randomized Controlled Trials: Evidence of Overinterpretation of Results
Source: Front Neurol. 2021 Jul 16;12:693937. doi: 10.3389/fneur.2021.693937 (PMC8322656; doi:10.3389/fneur.2021.693937)
Supplement: Supplementary file 3 [file Table_3.docx]

**Appendix 3. Adjustment of previously published spin criteria**

| **Original overinterpretation criteria by McGrath et al.** (5) | **Additional RCT-focused criteria by Boutron et al.** (3) | **Modified criteria used in this report** |
| --- | --- | --- |
| ﻿Positive conclusion, not reflecting the reported summary accuracy estimates |  | Conclusion, not reflecting the reported point estimate (and CI) of outcome |
| ﻿Positive conclusion, not taking high risk of bias and/or applicability concerns into account |  | *Discarded: lack of objectivity* |
| ﻿Positive conclusion, not taking heterogeneity into account |  | *Discarded: off topic* |
| ﻿Positive conclusion, focusing on the results of primary studies favoring the diagnostic accuracy of the test instead of the  metaanalysis results |  | *Discarded: off topic* |
| ﻿Positive conclusion, selectively focusing on a selection of subgroups, tests or accuracy estimates, while others were evaluated as well | Focus on statistically significant within-group comparison  Focus on statistically significant secondary outcomes  Focus on statistically significant subgroup analyses  Focus on statistically significant modified  population of analyses (eg, per-protocol analyses)  Focus on statistically significant within- and  between-group comparisons for secondary outcomes | Selectively focused conclusion:  Within group comparison  Secondary outcome  Subgroup analyses  Modified population of analyses  Focused on one arm while more arms were analyzed  Other |
| ﻿Positive conclusion, inappropriately extrapolated to a wider population or setting |  | Conclusion, inappropriately extrapolated to a wider population or setting |
| ﻿Positive conclusion, inappropriately extrapolated as surrogates for improvement in patient important outcomes |  | Conclusion, inappropriately extrapolated as surrogates for improvement in patient important outcomes |
| ﻿Stronger conclusion in abstract than full text |  | Stronger conclusion in abstract than full text |
| ﻿Conclusion claiming test equivalence or superiority based on indirect comparisons |  | *Discarded: off topic* |
| ﻿Conclusion claiming test equivalence or superiority without performing statistical comparisons; or claiming test equivalence for non-statistically significant results |  | *Discarded: off topic* |
|  |  | Linguistic spin |
| ﻿Intended role of index test in clinical pathway unclear |  | *Discarded: off topic* |
| ﻿No or inadequate assessment of risk of bias and applicability concerns |  | *Discarded: lack of discriminatory value* |
| ﻿Recommended statistical methods for metaanalysis performed |  | *Discarded: off topic* |
| ﻿Failure to report the number of studies and patients contributing to the meta- analyses in abstract |  | *Discarded: off topic* |
|  |  | No reporting of point estimate in abstract |
| ﻿No CIs around summary accuracy estimates in abstract |  | No CIs around point estimates of outcome in abstract |
|  |  | No P-value in abstract, in case of absence of CI in abstract |
|  |  | No SD in abstract, in case of absence of CI in abstract |
|  |  | No reporting of point estimate in full text |
| ﻿No CIs around summary accuracy estimates in full-text |  | No CIs around point estimates of outcome in full text |
|  |  | No P-value in full text, in case of absence of CI in abstract |
|  |  | No SD in full text, in case of absence of CI in abstract |
| ﻿No statistical assessment of heterogeneity performed |  | *Discarded: off topic* |
| ﻿No review limitations discussed |  | No study limitations discussed |
| ﻿Unclear conflict of interests |  | *Discarded: included in study characteristics* |
